# Mono- and di-acylated imidazolidine-2-thione derivatives: synthesis, cytotoxicity evaluation and computational studies

**DOI:** 10.1007/s11030-022-10487-5

**Published:** 2022-07-22

**Authors:** Anna Scarsi, Marco Ponassi, Chiara Brullo, Camillo Rosano, Andrea Spallarossa

**Affiliations:** 1 Department of Pharmacy, University of Genova, viale Benedetto XV, 3, 16132 Genoa, Italy; 2 Proteomics and Mass Spectrometry Unit, IRCCS Ospedale Policlinico San Martino, Largo R. Benzi 10, 16132 Genoa, Italy; 3 Present Address: IIT, Via Morego 30, 16163 Genoa, Italy

**Keywords:** Atypical Vilsmeier adduct, Acylation reaction, Acylthioureas, Cytotoxicity evaluation, Computational studies

## Abstract

Imidazolidine-2-thione substructure represents a pharmaceutically attractive scaffold, being included in different antimicrobial, anticancer and pesticide agents. To further evaluate the pharmaceutical potential of this chemical moiety, imidazolidine-2-thione was reacted with atypical Vilsmeier adducts, obtained by the condensation between dimethylacetamide and various acyl chlorides endowed with different electronic and steric properties. The formation of mono-acylated or di-acylated thiourea derivatives emerged to be affected by the nature of the considered acyl chloride reagent. Computational semi-empirical simulations were carried out to rationalize the relevant factor influencing the outcome of the reaction. As acylthioureas are pharmacologically relevant compounds, the chemical versatility of mono-acylated derivatives were evaluated by reacting benzoyl imidazolidin-2-thione with acyl chlorides. A small library of asymmetric di-acylthioureas was prepared and the obtained derivatives did not show any cytotoxicity on SKOV-3 and MCF-7 cancer cell lines. Additionally, in silico studies predicted good pharmacokinetics properties and promising drug-like characteristics for mono- and di-acylated thioureas. These considerations further support the value of the prepared compounds as interesting non-cytotoxic chemical scaffold useful in the medicinal chemistry field.

**Graphical abstract:**

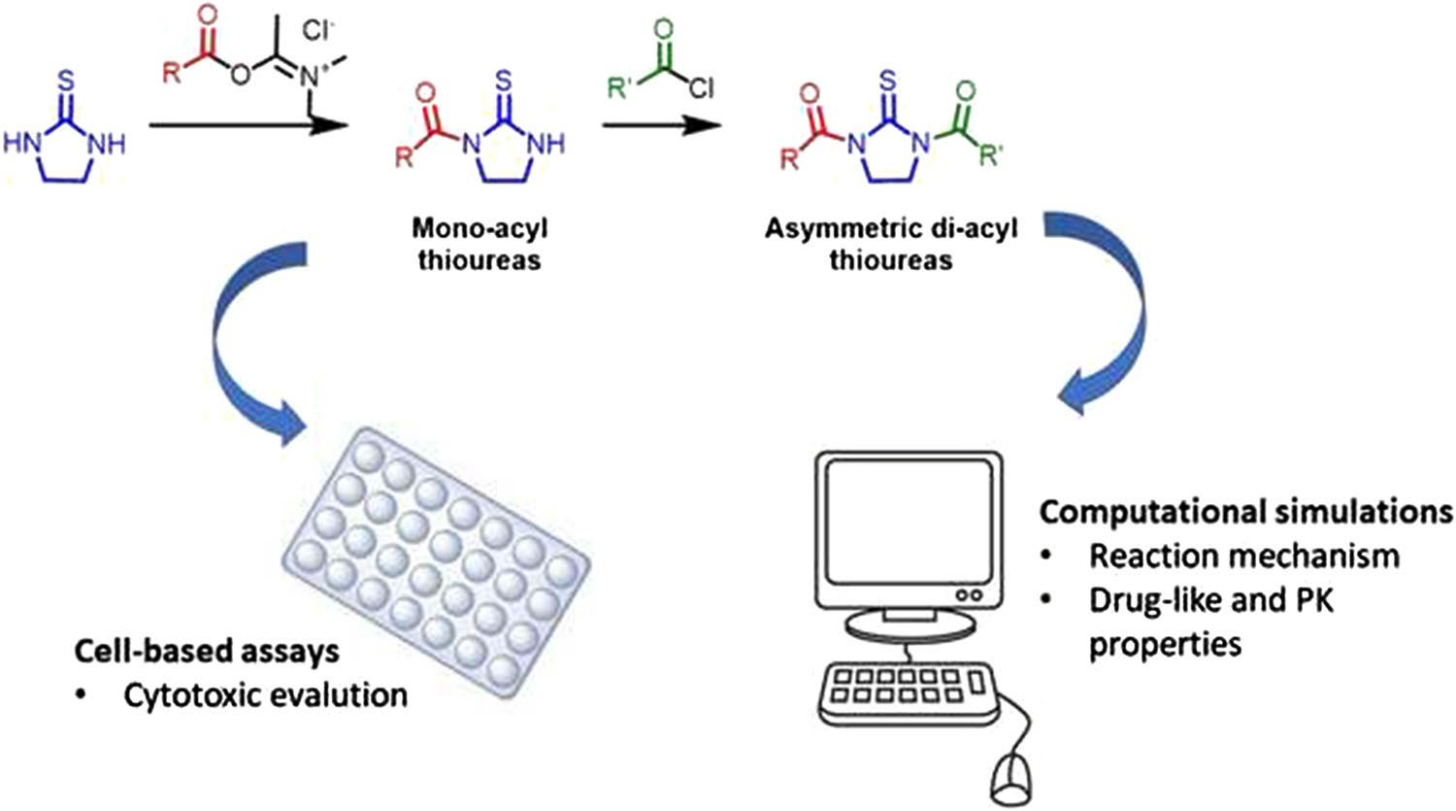

**Supplementary Information:**

The online version contains supplementary material available at 10.1007/s11030-022-10487-5.

## Introduction

Acylthioureas represent a class of pharmacologically relevant compounds endowed with a number of biological properties, including antiviral [1], antibacterial [2], antituberculosis [3, 4], anticonvulsant [5], antiplatelet [6–8], antiarrhythmic [8], analgesic [8], antihyperlipidemic [8], anaesthetic [8], thyrostatic [9] and antiproliferative [6–8, 10–12]. Imidazolidine-2-thione derivatives were found to be active as adenosine-A2B receptor antagonists with a relevant impact for treatment and/or prophylaxis of pulmonary and cardiovascular disorders and cancers [13], as well as GPR6 inverse agonists, an orphan receptor associated with neuropsychiatric disorders [14]. Furthermore, imidazolidine-2-thione analogues have been complexed with various metals (e.g. cadmium, zinc, silver, platinum) to obtain antimicrobial or anticancer agents [15–17]. This scaffold has also been used for the development of effective pesticides [18] and arthropod control agents [19].

**Fig. 1:**
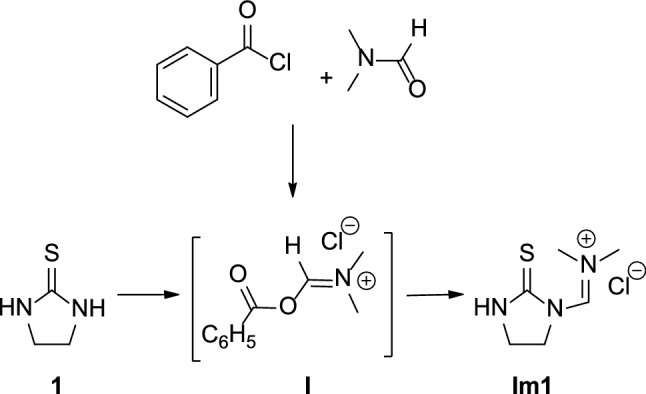
Formation of the key intermediate **Im1**

The biological activities of imidazolidine-2-thione derivatives [13–19] prompted us to investigate the condensation of **1** with the weak Vilsmeier reagent **I**, generated in situ through the reaction of *N*,*N*-dimethylformamide (DMF) and benzoyl chloride (Fig. 1) [20]. Adduct **I** is formed by reversible *O*-acylation of DMF [21–24] and proved to be a useful intermediate for the formylation of alcohols [25] and the synthesis of β-lactams [26]. Furthermore, the condensation of **1** with adduct **I** led to the isolation of *N*-methyleniminium salt **Im1** (Fig. 1) that was isolated and fully characterized by IR and NMR spectroscopy in our previous work [20]. **Im1** represented a key intermediate for the synthesis of pharmacologically relevant compounds endowed with antiproliferative, chelating and GPER-agonistic properties [27–29].

**Fig. 2:**
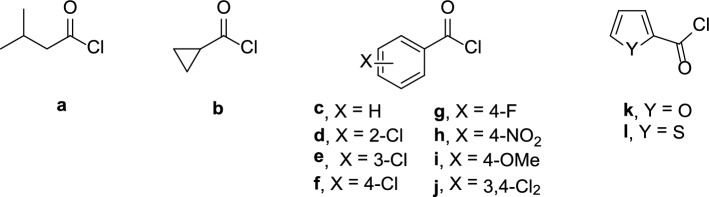
Chemical structures of acyl chlorides **a–l**

In order to extend our knowledge on the reactivity of weak Vilsmeier reagent towards cyclic thioureas, we studied the condensation of **1** with acyl chlorides **a-l** (Fig. 2) in the presence of *N*,*N*-dimethylacetamide (DMA), a DMF homologue. As reported in Fig. 2, the acyl chloride reagents included (cyclo)aliphatic (**a,b**), variously substituted benzoyl (**c–j**) and heteroaromatic substructures (**k,l**) to properly evaluate the effect of different electronic and steric properties on the reaction outcome.

## Results and discussion

### Reactivity of thiourea *1* with acyl chlorides

**Scheme 1:**
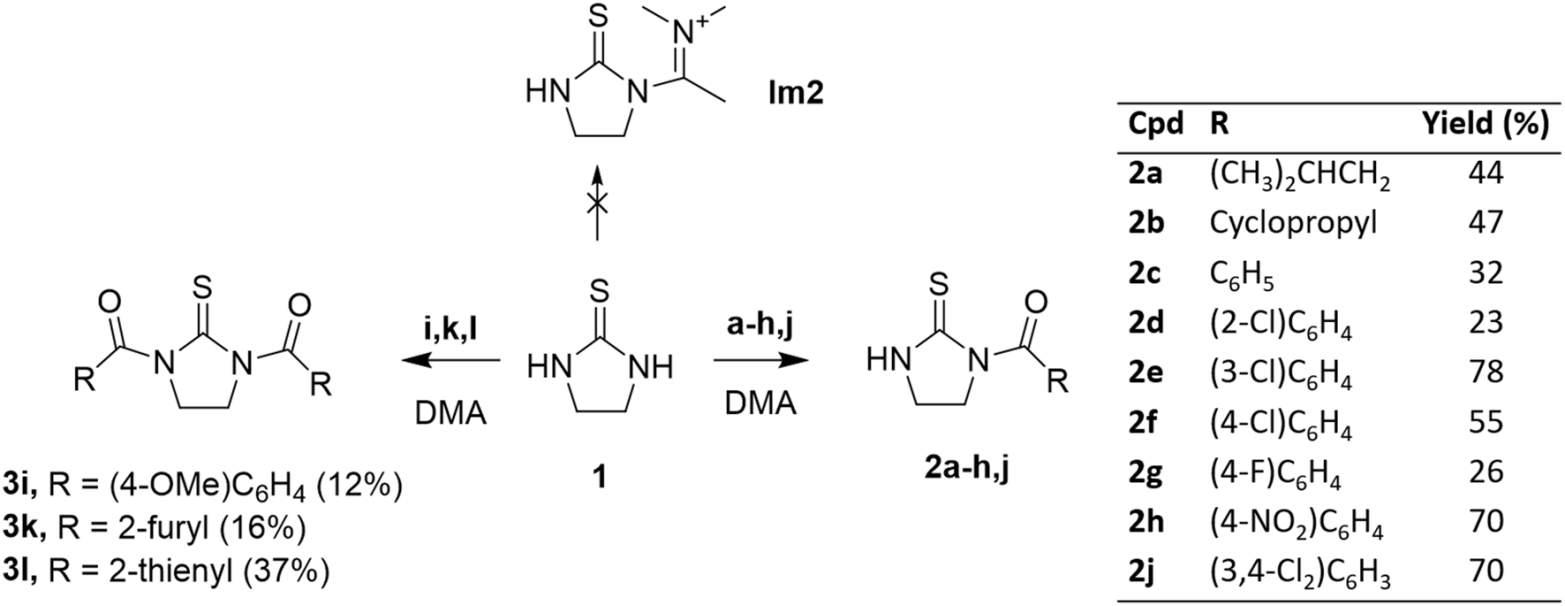
Reaction of cyclic thiourea with DMA and benzoyl chlorides

The replacement of DMF with its homologous DMA deeply affected the reaction outcome. Despite the in situ condensation of DMA and benzoyl chloride can afford the corresponding acyloxyiminium salt [21], the reaction of **1** with benzoyl chlorides **a–l** in DMA did not allow the isolation of iminium salt **Im2**, but led to the formation of mono- or di-acylthioureas (compounds **2** and **3**; Scheme 1), depending on the nature of the acylating agent. Thus, (cyclo)alkyl carbonyl chlorides, as well as benzoyl chlorides bearing electron withdrawing groups (i.e. halo or nitro groups), led to the formation of the mono-acylated derivatives **2a–h,j** in moderate-to-good yields (Scheme 1). Conversely, under the same reaction conditions, the condensation of **1** with one equivalent of 4-anisoyl chloride **i**, 2-furoyl chloride **k** and 2-thenoyl chloride **l** allowed the isolation of symmetric di-acylated thioureas **3i,k,l** in 12%, 16% and 37% yields, respectively (Scheme 1). According to the literature [30], the synthesis of mono-acylated thiourea derivatives is considered problematic, being the formation of the di-acylated compounds favoured also when acyl chlorides were used as limiting reagents. In fact, the only procedure reported in the literature for the synthesis of compound **2c** is based on a two-step intramolecular cyclization of 2-hydroxyethyl-thiocarbamides [31].

### Mechanistic considerations and hypothesis

**Scheme 2:**
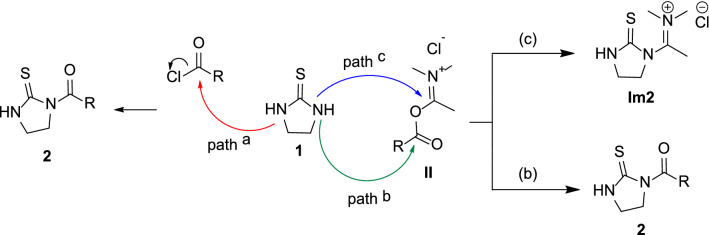
Possible reactions of **1** with different acylation species

In the tested synthetic conditions (i.e. acyl chlorides, DMA) thiourea **1** could react with two electrophilic species (namely, acyl chlorides and weak Vilsmeier reagents **II**) to form the mono-acylated compounds **2** (Scheme 2). In fact, as previously reported [21], the in situ condensation of DMA and benzoyl chloride led the formation of the corresponding acyloxyiminium salt that has been characterized by proton NMR and IR spectroscopy. The acyl chloride reagent could directly condense with **1** (path a, Scheme 2) or, as observed with DMF [20], react with DMA to afford the weak Vilsmeier reagents **II**. Out of the two electrophilic centres of intermediates **II** (i.e. ester carbonyl and iminium carbon), the nucleophilic nitrogen atom of **1** would selectively attack the ester carbonyl group (path b, Scheme 2), as the methyl group would hinder the iminium carbon preventing the formation of **Im2** (path c, Scheme 2).

To define whether thiourea acylation would occur via path (a) or (b) (Scheme 2), the calculated partial charges distribution (semi-empirical calculations, AM1 method) of acyl chlorides **d**, **f** and **i** was compared with that of the corresponding intermediates **II** (Fig. S30, Supporting information). The prediction indicated that the ester carbonyl of intermediates **II** would be more electrophilic than the acyl chloride carbonyl, thus suggesting **II** as the prevalent acylating species in the tested conditions.

**Scheme 3:**
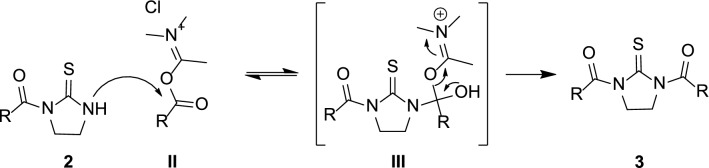
Hypothesized mechanism for the formation of di-acylthioureas **3**

As experimentally observed, the nature of acyl chloride reagents affects the acylation of thiourea **1** leading to the formation of compounds **2** or **3** (Scheme 3). The different reaction outcome would depend on the reactivity of **2** towards intermediates **II** (assumed to be the prevalent acylating species) in terms of (i) nucleophilicity of the mono-acylated compound or (ii) energetic content of intermediate **III** (Scheme 3).

**Fig. 3:**
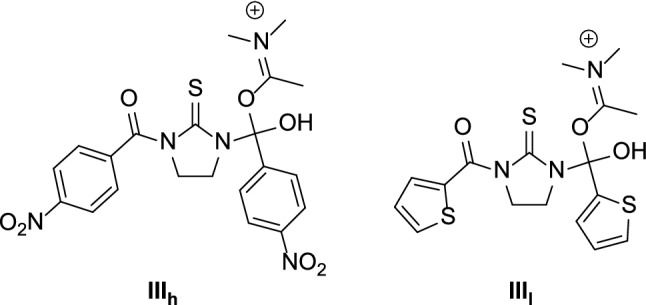
Chemical structures of intermediates **III**

Despite the condensation of **1** with acyl chlorides **h** and **l** led to different outcomes (namely, formation of mono- or di-acylthiourea compounds), the calculated partial charge of the thiourea nitrogen appears to be similar in the mono-acylated derivatives **2h** and **2l** (0.322 for **2h**; 0.323 for **2l**). Conversely, the calculated energy value of **III**_**h**_ (Fig. 3, *E* = 169.21 kcal/mol) was found to be significantly higher than that predicted for **III**_**l**_ (Fig. 3, *E* = 100.21 kcal/mol), thus suggesting that the stability of tetrahedral intermediate **III** would affect the outcome of the acylation reaction more than the nucleophilicity of the mono-acylated derivative.

### Synthesis of asymmetric di-acylated thioureas

**Scheme 4:**
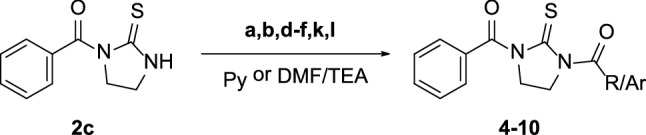
Synthesis of asymmetric di-acylthioureas **4–10**

The chemical accessibility to mono-acylated thioureas prompted us to evaluate their value as useful synthons for the preparation of unreported asymmetric di-acylthioureas. Thus, mono-benzoyl compound **2c** was selected as representative derivative of the mono-acylated series and condensed with acyl chlorides **a,b,d–f,k,l** endowed with different electronic and steric properties (Scheme 4). As detailed in Table 1, asymmetric di-acylthioureas **4–10** were obtained through two different synthetic protocols to take into consideration the different reactivity of acyl chlorides towards the mono-acylated thiourea. In particular, the acylation of **2c** with (hetero)aroyl chlorides **d–f,k,l** was carried out using pyridine as solvent as previously reported [27]. These experimental conditions proved to be ineffective for the preparation of derivatives **4** and **5** which were obtained by reacting **a** and **b** with **2c** in DMF in the presence of triethylamine (TEA). Compounds **4–10** were isolated in moderate-to-good yields, as detailed in Table 1.

**Table 1:**
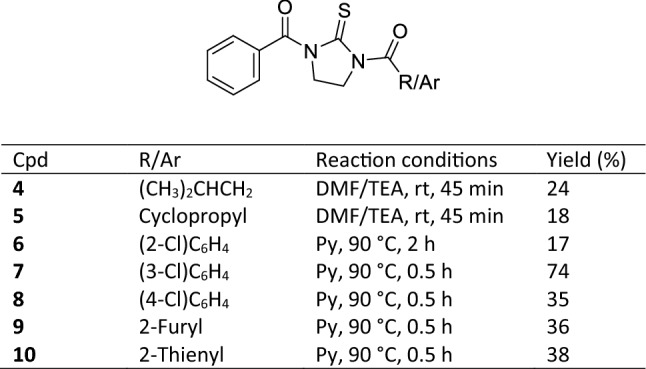
Reaction conditions and yields of compounds **4–10**

### Antiproliferative evaluation

The cytotoxic properties of acylthioureas **2c** and **4–10** were preliminary assessed against MCF-7 (breast cancer) and SKOV-3 (ovarian cancer) cell lines by MTT assay (Table 2). At the tested concentration (10 μM), all compounds were devoid of any antiproliferative activity against the selected cell lines, being the mean growth percentage values higher than 82.46%. These data supported the lack of cytotoxicity for the tested compounds and their potential value in different therapeutic areas, other than antitumor agents.

**Table 2:** Antiproliferative activity of compounds **2c, 4–10**

Cpd	Mean growth percentage (%)^a^	
	SKOV-3	MCF-7
**2c**	97.51	88.59
**4**	97.12	98.99
**5**	93.87	93.04
**6**	103.51	87.30
**7**	89.44	90.11
**8**	83.32	83.59
**9**	85.68	88.82
**10**	82.46	87.34

^a^Data mean values for three separate experiments. Variation among triplicate samples was less than 10%

### Pharmacokinetic properties and drug-likeness prediction

To evaluate the pharmaceutical relevance of the prepared derivatives, the pharmacokinetics properties and the drug-likeness of compounds **2–10** were calculated by SwissADME [32]. As detailed in Table 3, the calculated physicochemical parameters (i.e. Log*P* range: − 0.7 to + 5; MW range: 150–500 g/mol; TPSA range: 20–130 Å^2^; Fraction Csp3 range: 0.5–1; number of rotatable bonds: 0–9) indicated a good oral bioavailability for acylthioureas **2**. Furthermore, the predicted gastrointestinal (GI) absorption for the mono-acylated thioureas was high without any blood–brain barrier (BBB) penetration. According to the calculations, all derivatives **2** should not be able to inhibit cytochrome (CYP) isoforms 2C9, 2D6 and 3A4, whereas CYP1A2 would be blocked by (hetero)aroyl derivatives **2c–h, j** but not by the (cyclo)alkylcarbonyl compounds **2a,b**. Furthermore, the presence of a chlorine atom at position 4 of the benzoyl substructure would be related to the inhibition of CYP2C19 enzyme. The drug-like properties of derivatives **2** appear to be good, as no violations of the Lipinski rules were detected. Acylthioureas **2** did not showed any pan assay interference compound (PAINS) alerts, whereas the presence of the thiocarbonyl functionality (and of the nitro group for **2h**) was spotted as problematic fragment(s) according to the Brenk filters [33]. Noteworthily, this lead-likeness violation focussed on the physicochemical boundaries defining a good lead (i.e. a small and hydrophilic compound suitable for optimization) and does not undermine the pharmaceutical potential of the compound series. A molecular weight (MW) lower than 250 g/mol has been estimated to be a limitation of the lead-likeness (i.e. a suitability for optimization) of derivatives **2a–g**, as implemented by Teague and co-workers [34].

**Table 3:** Predicted pharmacokinetics and drug-like properties of mono-acylated thioureas **2**

	**2a**	**2b**	**2c**	**2d**	**2e**	**2f**	**2g**	**2h**	**2j**
*Physicochemical prop*									
MW (g/mol)	186.27	170.23	206.26	240.71	240.71	240.71	224.25	251.26	275.15
Fraction Csp^3^	0.75	0.71	0.2	0.2	0.2	0.2	0.2	0.2	0.2
Rotatable bonds	3	2	2	2	2	2	2	3	2
H-bond acceptors	1	1	1	1	1	1	2	3	1
H-bond donors	1	1	1	1	1	1	1	1	1
TPSA^a^ (Å^2^)	64.43	64.43	64.43	64.43	64.43	64.43	64.43	110.25	64.43
*Lipophilicity*									
LogP^b^	0.97	0.16	1.36	1.99	1.99	1.99	1.47	1.19	2.62
*Water solubility*									
Solubility (mg/ml)^c^	7.28	23.30	1.42	0.43	0.43	0.43	1.07	1.55	0.12
*Pharmacokinetics*									
GI absorption	High	High	High	High	High	High	High	High	High
BBB permeant	No	No	No	No	No	No	No	No	Yes
Pgp substrate	No	No	No	No	No	No	No	No	No
CYP1A2 inhibitor	No	No	Yes	Yes	Yes	Yes	Yes	Yes	Yes
CYP2C19 inhibitor	No	No	No	No	No	Yes	No	No	Yes
CYP2C9 inhibitor	No	No	No	No	No	No	No	No	No
CYP2D6 inhibitor	No	No	No	No	No	No	No	No	No
CYP3A4 inhibitor	No	No	No	No	No	No	No	No	No
*Drug-likeness*									
Lipinski violations	0	0	0	0	0	0	0	0	0
*Medicinal chemistry*									
PAINS alerts	0	0	0	0	0	0	0	0	0
Brenk alerts	1	1	1	1	1	1	1	3	1
Lead-likeness violations	1	1	1	1	1	1	1	0	0

^a^Topological polar surface area

^b^Predicted according to XLOGP3 program

^c^Values predicted by ESOL method [35]

According to the calculation carried out on di-acylthioureas **3–10** (Table 4), the physicochemical parameters (i.e. Log*P*; MW; TPSA; fraction Csp3 and number of rotatable bonds) of compounds **4** and **5** were considered suitable for oral bioavailability while the elevated degree of instauration (fraction Csp3 lower than 0.25) would negatively affect the oral absorption of derivatives **3,6–10**. Compounds **3–10** would act as inhibitors of different CYP isoforms (Table 4), being the 2C9 and 2C19 enzymes the most affected. Additionally, all compounds would be endowed with high GI absorption, whereas the penetration of the BBB would be related to the presence of a chloro-substituted benzoyl substructure. Likewise the mono-acylated precursor **2c**, compounds **3–10** displayed good drug-like properties as indicated by the absence of Lipinski violations. The di-acylated derivatives were predicted to be valuable lead compounds for further chemical optimization, despite the high log*P* values (greater than 3.5) of derivatives **6–8** and the MW value of **3i** exceeding the 350 g/mol cut-off. The presence of the thiocarbonyl functionality would represent a lead-likeness limitation, as highlighted by the Brenk alert value.

**Table 4:** Predicted pharmacokinetics and drug-like properties of di-acyl-thioureas **3–10**

	**3i**	**3k**	**3l**	**4**	**5**	**6**	**7**	**8**	**9**	**10**
*Physicochemical prop*										
MW (g/mol)	370.42	290.29	322.43	290.38	274.34	344.82	344.82	344.82	300.33	316.4
Fraction Csp^3^	0.21	0.15	0.15	0.4	0.36	0.12	0.12	0.12	0.13	0.13
Rotatable bonds	6	4	4	5	4	4	4	4	4	4
H-bond acceptors	4	4	2	2	2	2	2	2	3	2
H-bond donors	0	0	0	0	0	0	0	0	0	0
TPSA^a^ (Å^2^)	91.17	98.99	129.19	72.71	72.71	72.71	72.71	72.71	85.85	100.95
*Lipophilicity*										
Log*P*^b^	2.93	1.78	3.01	2.59	1.79	3.61	3.61	3.61	2.39	3.0
*Water solubility*										
Solubility (mg/ml)^c^	0.0437	0.394	0.0465	0.199	0.631	0.0146	0.0146	0.0146	0.14	0.0484
*Pharmacokinetics*										
GI absorption	High	High	High	High	High	High	High	High	High	High
BBB permeant	No	No	No	No	No	Yes	Yes	Yes	No	No
Pgp substrate	No	No	No	No	No	No	No	No	No	No
CYP1A2 inhibitor	No	Yes	Yes	No	Yes	No	No	No	No	Yes
CYP2C19 inhibitor	Yes	No	Yes	Yes	No	Yes	Yes	Yes	Yes	Yes
CYP2C9 inhibitor	Yes	No	Yes	No	No	Yes	Yes	Yes	No	Yes
CYP2D6 inhibitor	No	No	No	No	No	No	No	No	No	No
CYP3A4 inhibitor	Yes	No	No	No	No	No	No	No	No	No
*Drug-likeness*										
Lipinski violations	0	0	0	0	0	0	0	0	0	0
*Medicinal chemistry*										
PAINS alerts	0	0	0	0	0	0	0	0	0	0
Brenk alerts	1	1	1	1	1	1	1	1	1	1
Lead-likeness violations	1	0	0	0	0	1	1	1	0	0

^a,b,c^See Table 3

## Conclusions

The condensation of cyclic thiourea **1** with acyl chlorides in DMA was identified as a novel, single-step, effective procedure for the preparation of mono-acylated derivatives **2**. The developed procedure emerged to be versatile and allowed the preparation of mono (cyclo)alkyl carbonyl, benzoyl and heteroaryl thiourea derivatives. The identified conditions led to the synthesis of mono-acylated imidazolidine-2-thione compounds whose preparation proved to be difficult in other conditions reported in the literature; in fact, the mono-acyl derivatives emerged to be more reactive than the parent thioureas towards the acylating agent. The chemical accessibility to mono-benzoyl thiourea **2c** allowed the preparation of a small library of unreported asymmetric di-acylthioureas. The obtained derivatives **4–10** were devoid of any cytotoxicity in preliminary MTT screening carried out against MCF-7 and SKOV-3 cancer cells. The lack of cytotoxicity represents the starting point for future studies focussed on the evaluation of the pharmaceutical potentials of these compounds in therapeutic areas other than anticancer agents. Furthermore, in silico studies predicted for mono- and di-acylated thioureas good drug-like and pharmacokinetics properties that further support the potential of compounds **2–10** in the medicinal chemistry area.

## Experimental section

### Chemistry

Commercially available thiourea **1** and acyl chlorides **a-l** were purchased by Alfa-Aesar and Sigma-Aldrich. DMF, DMA and pyridine were reagent grade and were dried on molecular sieves (5 Å 1/16" inch pellets). Unless otherwise stated, all commercial reagents were used without further purification. Organic solutions were dried over anhydrous sodium sulphate. Thin-layer chromatography (TLC) system for routine monitoring the course of parallel reactions and confirming the purity of analytical samples employed aluminium-backed silica gel plates (Merck DC-Alufolien Kieselgel 60 F254). DCM or DCM/methanol (9:1) were used as a developing solvent and detection of spots was made by UV light and/or by iodine vapours. Melting points were determined on a Fisher-Johns apparatus and are uncorrected. ^1^H NMR and ^13^C NMR spectra were recorded on a Varian Gemini, Bruker DPX-300 or JEOL JNM-ECZR instrument; chemical shifts were reported in d (ppm) units relative to the internal reference tetramethylsilane and the splitting patterns were described as follows: s (singlet), bs (broad singlet), d (doublet), t (triplet), q (quartet) and m (multiplet). The first-order values reported for coupling constants J were given in Hz. Elemental analyses were performed by an EA1110 Analyzer, Fison Instruments (Milan).

### Synthesis of compounds 2 and 3

A dry DMA (8 ml) solution of **1** (1.04 g, 10 mmol) and the proper acyl chloride (10 mmol) were stirred at 90 °C for 30 min. After cooling to rt, water (30 ml) and 1 M K_2_CO_3_ solution (pH 8) were added. The mixture was extracted with DCM (2 × 15 ml) and the pooled organic phases were washed with water (1 × 10 ml), dried with anhydrous Na_2_SO_4_ and concentrated under reduced pressure. The crude material was purified by crystallization from the proper solvent or solvent mixture.

#### 3-Methyl-1-(2-thioxoimidazolidin-1-yl)butan-1-one (2a)

White solid; yield 44%; mp: 135–137 °C (DCM/MeOH). ^1^H NMR (400 MHz, DMSO-D_6_): *δ* 0.90 (d, *J* = 6.6 Hz, 6H, 2 × CH_3_); 2.04–2.19 (m, 1H, CH); 3.20 (d, *J* = 6.9 Hz, 2H, CH_2_–CO); 3.40–3.49 (m, 2H, CH_2_N); 3.95–4.04 (m, 2H, CH_2_N); 9.66 (bs, 1H, NH deuterable). ^13^C NMR (101 MHz, DMSO-D_6_): *δ* 22.3, 25.2, 44.8, 46.9, 173.1, 179.2. Anal. Calcd for C_8_H_14_N_2_OS: C, 51.58; H, 7.58; N, 15.04; S, 17.21. Found: C, 51.28; H, 7.52; N, 15.14; S; 17.23.

#### Cyclopropyl(2-thioxoimidazolidin-1-yl)methanone (2b)

White solid; yield: 47%; mp: 143–145 °C (DCM/MeOH). ^1^H NMR (400 MHz, DMSO-D_6_): *δ* 0.88–0.97 (m, 4H, 2 × CH_2_ cycloprop.); 3.41–3.51 (m, 2H, CH_2_N); 3.94–4.03 (m, 2H, CH_2_N); 4.10–4.21 (m, 1H, CHCO); 9.73 (bs, 1H, NH exchangeable). ^13^C NMR (101 MHz, DMSO-D_6_): *δ* 10.2, 13.5, 40.3, 47.3, 174.7, 179.5. Anal. Calcd for C_7_H_10_N_2_OS: C, 49.39; H, 5.98; N, 16.45; S, 18.83. Found: C, 49.33; H, 5.69; N, 16.53; S, 18.66.

#### Phenyl(2-thioxoimidazolidin-1-yl)methanone (2c)

Yellow solid; yield 32%; mp: 152–154 °C (neat DCM). ^1^H NMR (400 MHz, DMSO-D_6_): *δ* 3.54–3.63 (m, 2H, CH_2_N); 4.06–4.15 (m, 2H, CH_2_N); 7.32–7.41 (m, 2H, arom. H); 7.43–7.55 (m, 2H, arom. H); 9.74 (bs, 1H, NH deuterable). ^13^C NMR (101 MHz, DMSO-D_6_): *δ* 41.2, 48.1, 127.4, 128.8, 131.0, 135.7, 171.2, 180.1. Anal. Calcd for C_10_H_10_N_2_OS: C, 58.23; H, 4.89; N, 13.58; S, 15.55. Found: C, 58.41; H, 4.70; N, 13.77; S, 15.35.

#### (2-chlorophenyl)(2-thioxoimidazolidin-1-yl)methanone (2d)

White solid; yield 23%; mp: 195–197 °C (DCM/MeOH). ^1^H NMR (400 MHz, DMSO-D_6_): 3.54–3.63 (m, 2H, CH_2_N); 4.12–4.21 (m, 2H, CH_2_N); 7.27–7.44 (m, 4H, arom. H); 9.82 (bs, 1H, NH deuterable). ^13^C NMR (101 MHz, DMSO-D_6_): *δ* 40.9, 46.5, 126.6, 128.7, 129. 8, 130.5, 136.5, 167.1, 178.7. Anal. Calcd for C_10_H_9_ClN_2_OS: C, 49.90; H, 3.77; N, 11.64; S, 13.32. Found: C, 49.85; H, 3.68; N, 11.68; S, 13.42.

#### (3-chlorophenyl)(2-thioxoimidazolidin-1-yl)methanone (2e)

White solid: yield 78%; mp: 134–137 °C (DCM/MeOH). ^1^H NMR (400 MHz, DMSO-D_6_): *δ* 3.54–3.63 (m, 2H, CH_2_N); 4.06–4.15 (m, 2H, CH_2_N); 7.35–7.61 (m, 4H, arom. H); 9.84 (bs, 1H, NH deuterable). Anal. Calcd for C_10_H_9_ClN_2_OS: C, 49.90; H, 3.77; N, 11.64; S, 13.32. Found: C, 50.27; H, 3.82; N, 11.86; S, 13.53.

#### (4-chlorophenyl)(2-thioxoimidazolidin-1-yl)methanone (2f)

Yellow solid; yield 55%; mp: 155–157 °C (DCM/MeOH). ^1^H NMR (400 MHz, DMSO-D_6_): *δ* 3.54–3.62 (m, 2H, CH_2_N); 4.06–4.16 (m, 2H, CH_2_N); 7.41–7.47 (m, 2H, arom. H); 7.50–7.56 (m, 2H, arom. H); 9.80 (bs, 1H, NH deuterable). Anal. Calcd for C_10_H_9_ClN_2_OS: C, 49.90; H, 3.77; N, 11.64; S, 13.32. Found: C, 50.08; H, 3.40; N, 11.53; S, 13.06.

#### (4-fluorophenyl)(2-thioxoimidazolidin-1-yl)methanone (2g)

White solid; yield 26%; mp: 165–167 °C (DCM/MeOH). ^1^H NMR (400 MHz, DMSO-D_6_): *δ* 3.53–3.62 (m, 2H, CH_2_N); 4.06–4.14 (m, 2H, CH_2_N); 7.15–7.25 (m, 2H, arom. H); 7.54–7.64 (m, 2H, arom. H); 9.77 (bs, 1H, NH, deuterable). Anal. Calcd for C_10_H_9_N_2_OSF: C, 53.56; H, 4.05; N, 12.49; S, 14.30. Found: C, 53.53; H, 4.05; N, 12.67; S, 14.59.

#### (4-Nitrophenyl)(2-thioxoimidazolidin-1-yl)methanone (2h)

Yellow solid; yield 70%; mp: 179–181 °C (DCM/MeOH). ^1^H NMR (200 MHz, CDCl_3_): *δ* 3.57–3.70 (m, 2H, CH_2_N); 4.08–4.278 (m, 2H, CH_2_N); 7.67–7.78 and 8.17–8.29 (m, 4H, arom. H); 9.97 (bs, 1H, NH, deuterable). Anal. Calcd for C_10_H_9_N_3_O_3_S: C, 47.80; H, 3.61; N, 16.72; S, 12.76. Found: C, 47.50; H, 3.50; N, 16.62; S, 12.52.

#### (3,4-Dichlorophenyl)(2-thioxoimidazolidin-1-yl)methanone (2j)

White solid; yield 70%; mp: 150–155 °C (DCM/MeOH). ^1^H NMR (400 MHz, DMSO-D_6_): *δ* 3.45–3.64 (m, 2H, CH_2_N); 4.06–4.15 (m, 2H, CH_2_N); 7.43–7.50 (m, 1H, arom. H); 7.62–7.68 (m, 1H, arom. H); 7.75–7.80 (m, 1H, arom. H); 9.91 (bs, 1H, NH, deuterable). ^13^C NMR (101 MHz, DMSO-D_6_): *δ* 41.3, 47.8, 128.6, 129.9, 130.1, 130.6, 133.2, 136.3, 168.6, 179.6. Anal. Calcd for C_10_H_8_N_2_Cl_2_OS: C, 43.65; H, 2.93; N, 10.18; S, 11.65. Found: C, 43.44; H, 2.91; N, 10.44; S, 11.82.

#### (2-Thioxoimidazolidine-1,3-diyl)bis((4-methoxyphenyl)methanone) (3i)

Yellow solid; yield 12%; mp: 198–201 °C (DCM/MeOH). ^1^H NMR (400 MHz, CDCl_3_): *δ* 3.83 (s, 6H, 2 × OCH_3_); 4.16–4.20 (m, 4H, 2 × CH_2_N); 6.81–6.90 (m, 4H, arom. H); 7.65–7.72 (m, 4H, arom. H). Anal. Calcd for C_19_H_18_N_2_O_4_S: C, 61.61; H, 4.90; N, 7.56; S, 8.65. Found: C, 61.81; H, 4.79; N, 7.62; S, 8.75.

#### (2-Thioxoimidazolidine-1,3-diyl)bis(furan-2-ylmethanone) (3k)

Yellow solid; yield 16%; mp: 141–144 °C (DCM/MeOH). ^1^H NMR (200 MHz, CDCl_3_): *δ* 4.07- 4.23 (m, 4H, 2 × CH_2_N); 6.42–6.58 (m, 2H, H(4)-furan); 7.12–7.25 (m, 2H, H(3)-furan); 7.43–7.60 (m, 2H, H(5)-furan). Anal. Calcd for C_13_H_10_N_2_O_4_S: C, 53.79; H, 3.47; N, 9.65; S, 11.04. Found: C, 53.70; H, 3.47; N, 9.81; S, 11.36.

#### (2-Thioxoimidazolidine-1,3-diyl)bis(thiophen-2-ylmethanone) (3l)

Yellow solid; yield 37%; mp: 195–197 °C (DCM/MeOH). ^1^H NMR (400 MHz, DMSO-D_6_): *δ* 4.18–4.21 (m, 4H, 2 × CH_2_N); 7.17–7.24 (m, 2H, H4-thiophene); 7.86–7.94 (m, 2H, 2 × H5-thiophene); 7.98–8.05 (m, 2H, 2 × H3-thiophene). ^13^C NMR (101 MHz, DMSO-D_6_): *δ* 47.1, 128.1, 134.9, 135.4, 136.8, 164.6, 179.5. Anal. Calcd for C_13_H_10_N_2_O_2_S_3_: C, 48.43; H, 3.13; N, 8.69; S, 29.83. Found: C, 48.97; H, 3.10; N, 8.74; S, 29.62.

### Synthesis of compounds 4 and 5

To a dry DMF (10 ml) solution of **2c** (0.35 g, 1.7 mmol), TEA (266 ml, 1.9 mmol) and the proper acyl chloride (1.9 mmol) were added sequentially. The reaction mixture was stirred at rt for 45 min and then heated at 40 °C for 15 min. After dilution with water (40 ml) the mixture was kept at 4 °C for 2 hours and the precipitated solid was filtered. The crude material was purified by crystallization from DCM/MeOH mixture.

#### 1-(3-Benzoyl-2-thioxoimidazolidin-1-yl)-3-methylbutan-1-one (4)

Yellow solid; yield 24%; mp: 100–103 °C. ^1^H NMR (400 MHz, CDCl_3_): *δ* 0.92–1.01 (m, 6H, 2 × CH_3_); 2.16–2.30 (m, 1H, CH); 3.09–3.16 (m, 2H, CH_2_–CO); 4.00–4.10 (m, 2H, CH_2_N); 4.11–4.22 (m, 2H, CH_2_N); 7.36–7.46 (m, 2H, arom. H); 7.48–7.55 (m, 1H, arom. H); 7.62–7.69 (m, 2H, arom. H). ^13^C NMR (101 MHz, CDCl_3_): *δ* 22.6, 25.2, 44.6, 44.8, 47.1, 128.3, 129.2, 132.4, 134.8, 172.4, 174.6, 178.6. Anal. Calcd for C_15_H_18_N_2_O_2_S: C, 62.04; H, 6.25; N, 9.65; S, 11.04. Found: C, 61.86; H, 6.05; N, 9.84; S, 11.07.

#### (3-Benzoyl-2-thioxoimidazolidin-1-yl)(cyclopropyl)methanone (5)

White solid; yield 18%; mp: 136–137 °C. ^1^H NMR (300 MHz, CDCl_3_): *δ* 0.99–1.04 (m, 2H, CH_2_cyc); 1.18–1.21 (m, 2H, CH_2_cyc); 3.67–3.70 (m, 1H, CHCO); 4.07–4.14 (m, 4H, 2 × CH_2_N); 7.39–7.68 (m, 5H, arom. H). Anal. Calcd for C_14_H_14_N_2_O_2_S: C, 61.29; H, 5.14; N, 10.21; S, 11.68. Found: C, 61.32; H, 4.91; N, 10.20; S, 12.11.

### Synthesis of compounds 6–10

An anhydrous pyridine (10 ml) solution of **2c** (0.325 g, 1.57 mmol) and the proper acyl chloride (1.9 mmol) were heated at 90 °C for 0.5 h (for 6, 2 h). After dilution with water (40 ml) the mixture was kept at 4 °C for 2 h and the precipitated solid was filtered. The crude material was purified by crystallization from DCM/MeOH mixture.

#### (3-Benzoyl-2-thioxoimidazolidin-1-yl)(2-chlorophenyl)methanone (6)

Yellow solid; yield 16%; mp: 131–133 °C. ^1^H NMR (400 MHz, DMSO-D_6_): *δ* 4.31–4.38 (m, 4H, 2 × CH_2_N); 7.44–7.53 (m, 7H, arom. H); 7.59–7.62 (m, 2H, arom. H). Anal. Calcd for C_17_H_13_N_2_O_2_SCl: C, 59.21; H, 3.80; N, 8.12; S, 9.30. Found: C, 59.34; H, 3.78; N, 7.99; S, 10.01.

#### (3-Benzoyl-2-thioxoimidazolidin-1-yl)(3-chlorophenyl)methanone (7)

Ivory solid; yield 74%; mp: 150–151 °C. ^1^H NMR (200 MHz, CDCl_3_): *δ* 4.13–4.30 (m, 4H, 2 × CH_2_N); 7.25–7.68 (m, 9H, arom. H). Anal. Calcd for C_17_H_13_ClN_2_O_2_S: C, 59.21; H, 3.80; N, 8.12; S, 9.30. Found: C, 59.25; H, 4.02; N, 8.35; S, 8.52.

#### (3-Benzoyl-2-thioxoimidazolidin-1-yl)(4-chlorophenyl)methanone (8)

Yellow solid; yield 35%; mp: 201–203 °C. ^1^H NMR (400 MHz, CDCl_3_): *δ* 4.21–4.25 (m, 4H, 2 × CH_2_N); 7.29–7.64 (m, 9H, arom. H). ^13^C NMR (101 MHz, CDCl_3_): *δ* 45.6, 128.2, 128.5, 129.1, 130.6, 132.4, 132.9, 134.4, 138.5, 171.1, 172.0, 178.9. Anal. Calcd for C_17_H_13_ClN_2_O_2_S: C, 59.21, H, 3.80, N, 8.21, S, 9.30. Found: C, 60.86, H, 3.75, N, 8.65, S, 8.16.

#### (3-Benzoyl-2-thioxoimidazolidin-1-yl)(furan-2-yl)methanone (9)

Yellow solid; yield 36%; mp: 175–176 °C. ^1^H NMR (400 MHz, CDCl_3_): *δ* 4.24–4.29 (m, 4H, 2 × CH_2_N); 6.55–6.58 (m, 1H, H furan); 7.25–7.72 (m, 7H, Arom. H + H furan). ^13^C NMR (101 MHz, CDCl_3_): *δ* 45.5, 46.0, 112.7, 119.9, 128.2, 129.1, 132.3, 134.6, 145.9, 147.1, 160.5, 171.9, 178.4. Anal. Calcd for C_15_H_12_N_2_O_3_S: C, 59.99; H, 4.03; N, 9.33; S, 10.68. Found: C, 59.86; H, 4.25; N, 9.59; S, 9.18.

#### (3-Benzoyl-2-thioxoimidazolidin-1-yl)(thiophen-2-yl)methanone (10)

Yellow solid; yield 38%; mp: 186–188 °C. ^1^H NMR (400 MHz, CDCl_3_): *δ* 4.14–4.31 (m, 4H, 2 × CH_2_N); 6.98–7.09 (m, 1H, H thiophen); 7.31–7.75 (m, 7H, arom. H and H thiophen). ^13^C NMR (101 MHz, CDCl_3_): *δ* 45.8, 46.1, 127.6, 128.1, 129.1, 132.3, 133.8, 134.6, 134.9, 136.8, 165.1, 172.0, 179.3. Anal. Calcd for C_15_H_12_N_2_O_2_S_2_: C, 56.94; H, 3.82; N, 8.85; S, 20.27. Found: C, 56.95; H, 4.12; N, 9.00; S, 18.19.

### Biology

To perform MTT assay, SKOV-3 (ovarian adenocarcinoma, ATCC, Manassas, VA) and MCF-7 (breast adenocarcinoma, Biologic Bank and Cell Factory, IRCCS Policlinico San Martino, Genoa, Italy) cell lines were cultured in DMEM (added with 10% FBS, 2 mM Glutamine and 1% penstrep. Reagents were acquired from EuroClone, Milan, Italy) and incubated in humidified conditions at 37 °C with 5% CO_2_. Chemical compounds were dissolved in DMSO to give a 10 mM stock solution. Then, once diluted in growth medium, they were added to the cultured cells at a final working concentration of 10 μM and incubated for 48 h. At the end of the incubation, 30 μl of MTT (3-(4,5-dimethyl-2-thiazolyl)-2,5-diphenyl-2H-tetrazolium bromide) at a concentration of 2 mg/ml in PBS were added in each well and incubated 4 h. Finally, the surnatant was removed and 100 μl/well of DMSO were added to dissolve the Formazan precipitate. After 20 min, the results were read at 570 nm. Results are expressed as percentage of the control samples where cells have been treated with the same amount of DMSO but without any chemical compound. The assay was repeated three times and a single compound was tested six times. Means and standard deviations were calculated.

### Computational calculations

The chemical structures of studied compounds were drawn with MOE2009.10 (builder module) and energy minimization was carried out according to AM1, as implemented in MOE software. The calculations were run on a Linux PC (Intel® processor Core™ i7-2600 CPU@3.40 GHz).

## Supplementary Information

Below is the link to the electronic supplementary material.

Supplementary file1 (DOCX 9061 kb)
