# Supplementary material for: Mono- and di-acylated imidazolidine-2-thione derivatives: synthesis, cytotoxicity evaluation and computational studies
Source: Mol Divers. 2022 Jul 22;27(3):1285–95. doi: 10.1007/s11030-022-10487-5 (PMC10276106; doi:10.1007/s11030-022-10487-5)
Supplement: Supplementary file 1 — Supplementary file1 (DOCX 9061 kb) [file 11030_2022_10487_MOESM1_ESM.docx]

Mono- and diacylated imidazolidine-2-thione derivatives: synthesis, antiproliferative activity and computational studies

Anna Scarsi ^1,†^, Marco Ponassi ^2^, Chiara Brullo ^1^, Camillo Rosano ^2^ and Andrea Spallarossa ^1,^*

^1^ Department of Pharmacy, University of Genova, viale Benedetto XV, 3, 16132, Genova, Italy; anna.scarsi@iit.it (A.S.); chiara.brullo@unige.it (C.B.).

^2^ Proteomics and Mass Spectrometry Unit, IRCCS Ospedale Policlinico San Martino, Largo R. Benzi 10, 16132, Genova, Italy; marco.ponassi@hsanmartino.it (M.P.); camillo.rosano@hsanmartino.it (C.R.).

† Current address: IIT, Via Morego, 30, 16163 Genova, Italy

***** Correspondence: andrea.spallarossa@unige.it

**Index**

**Fig. S1** ^1^H NMR (400 MHz, DMSO-D_6_) of compound **2a**

**Fig. S2** ^13^C NMR (101 MHz, DMSO-D_6_) of compound **2a**

**Fig. S3** ^1^H NMR (400 MHz, DMSO-D_6_) of compound **2b**

**Fig. S4** ^13^C NMR (101 MHz, DMSO-D_6_) of compound **2b**

**Fig. S5** ^1^H NMR (400 MHz, DMSO-D_6_) of compound **2c**

**Fig. S6** ^13^C NMR (101 MHz, DMSO-D_6_) of compound **2c**

**Fig. S7** ^1^H NMR (400 MHz, DMSO-D_6_) of compound **2d**

**Fig. S8** ^13^C NMR (101 MHz, DMSO-D_6_) of compound **2d**

**Fig. S9** ^1^H NMR (400 MHz, DMSO-D_6_) of compound **2e**

**Fig. S10** ^1^H NMR (400 MHz, DMSO-D_6_) of compound **2f**

**Fig. S11** ^1^H NMR (400 MHz, DMSO-D_6_) of compound **2g**

**Fig. S12** ^1^H NMR (200 MHz, DMSO-D_6_) of compound **2h**

**Fig. S13** ^1^H NMR (400 MHz, DMSO-D_6_) of compound **2j**

**Fig. S14** ^13^C NMR (101 MHz, DMSO-D_6_) of compound **2j**

**Fig. S15** ^1^H NMR (400 MHz, CDCl_3_) of compound **3i**

**Fig. S16** ^1^H NMR (200 MHz, CDCl_3_) of compound **3k**

**Fig. S17** ^1^H NMR (400 MHz, DMSO-D_6_) of compound **3l**

**Fig. S18** ^13^C NMR (101 MHz, DMSO-D_6_) of compound **3l**

**Fig. S19**^1^H NMR (400 MHz, CDCl_3_) of compound **4**

**Fig. S20** ^13^C NMR (101 MHz, CDCl_3_) of compound **4**

**Fig. S21** ^1^H NMR (300 MHz, CDCl_3_) of compound **5**

**Fig. S22** ^1^H NMR (400 MHz, DMSO-D_6_) of compound **6**

**Fig. S23** ^1^H NMR (200 MHz, CDCl_3_) of compound **7**

**Fig. S24** ^1^H NMR (400 MHz, CDCl_3_) of compound **8**

**Fig. S25** ^13^C NMR (101 MHz, CDCl_3_) of compound **8**

**Fig. S26** ^1^H NMR (400 MHz, CDCl_3_) of compound **9**

**Fig. S27** ^13^C NMR (101 MHz, CDCl_3_) of compound **9**

**Fig. S28** ^1^H NMR (400 MHz, CDCl_3_) of compound **10**

**Fig. S29** ^13^C NMR (101 MHz, CDCl_3_) of compound **10**

**Fig. S30** Partial charge distribution of benzoyl chloride (A), 4-chlorobenzoyl chloride (B), 4-anisoyl chloride (C) and their corresponding intermediates II (D-F).

**Fig. S1** ^1^H NMR (400 MHz, DMSO-D_6_) of compound **2a**

**Fig. S2** ^13^C NMR (101 MHz, DMSO-D_6_) of compound **2a**

**Fig. S3** ^1^H NMR (400 MHz, DMSO-D_6_) of compound **2b**

**Fig. S4** ^13^C NMR (101 MHz, DMSO-D_6_) of compound **2b**

**Fig. S5** ^1^H NMR (400 MHz, DMSO-D_6_) of compound **2c**

**Fig. S6** ^13^C NMR (101 MHz, DMSO-D_6_) of compound **2c**

**
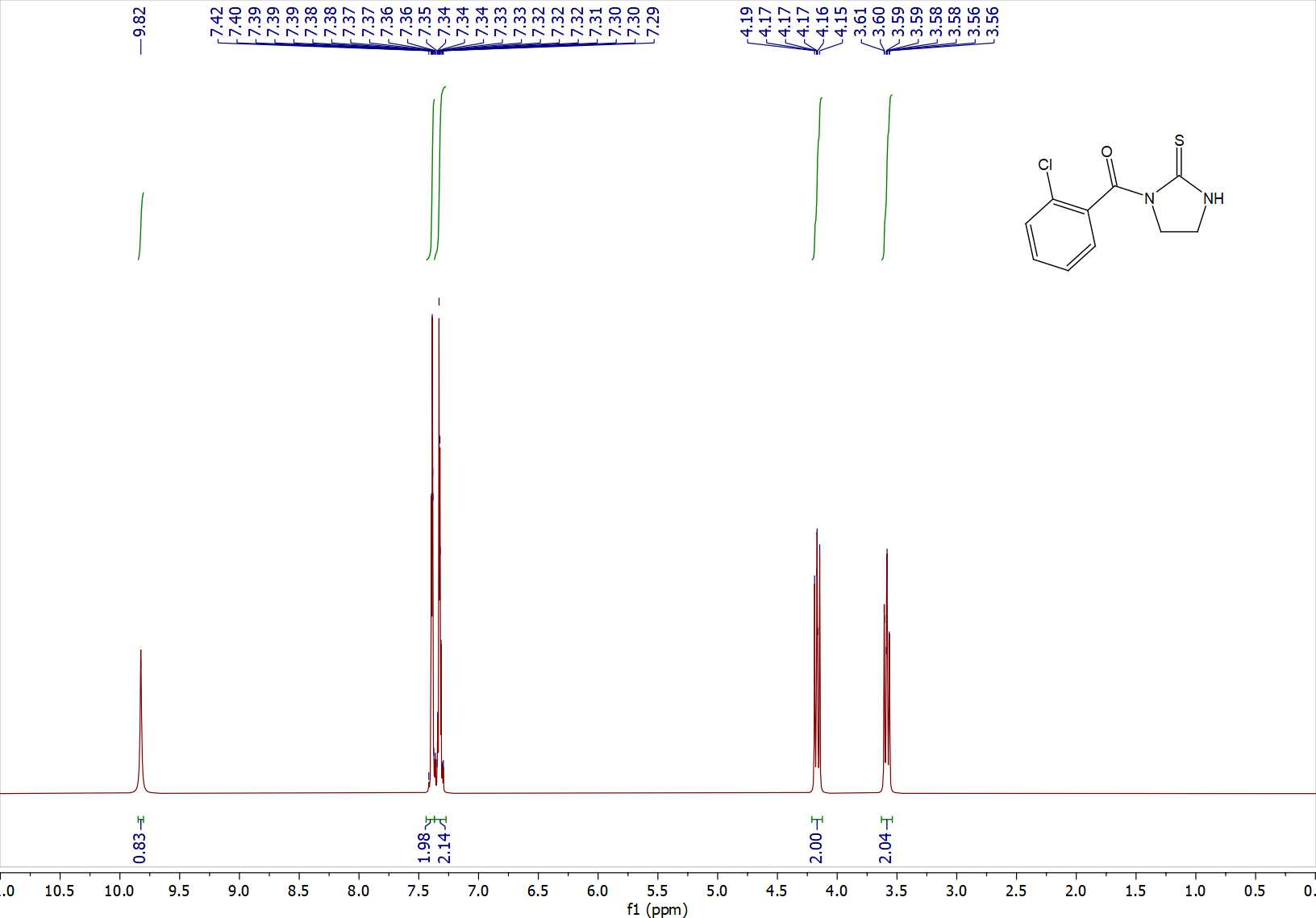
**

**Fig. S7** ^1^H NMR (400 MHz, DMSO-D_6_) of compound **2d**

**
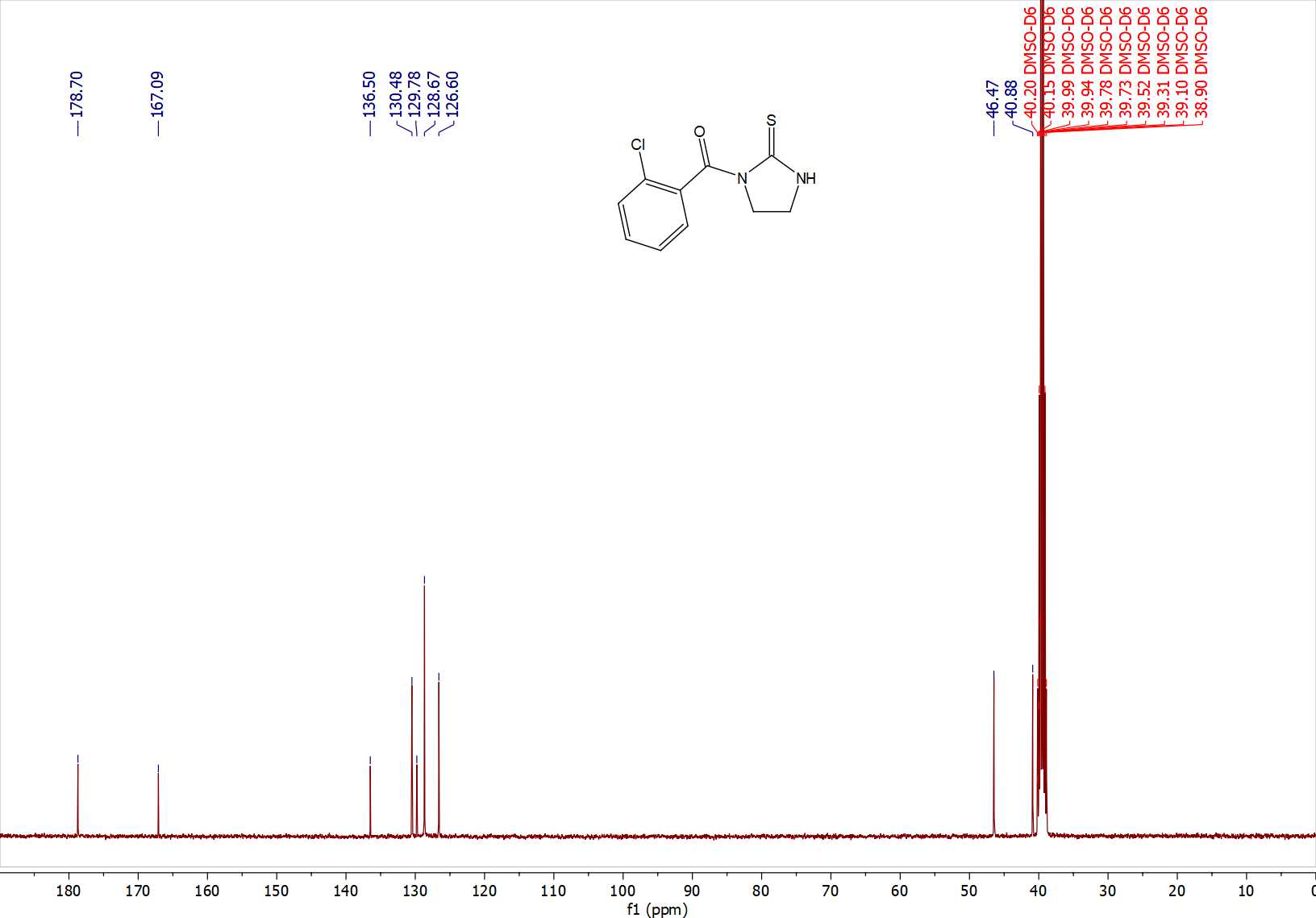
**

**Fig. S8** ^13^C NMR (101 MHz, DMSO-D_6_) of compound **2d**

**
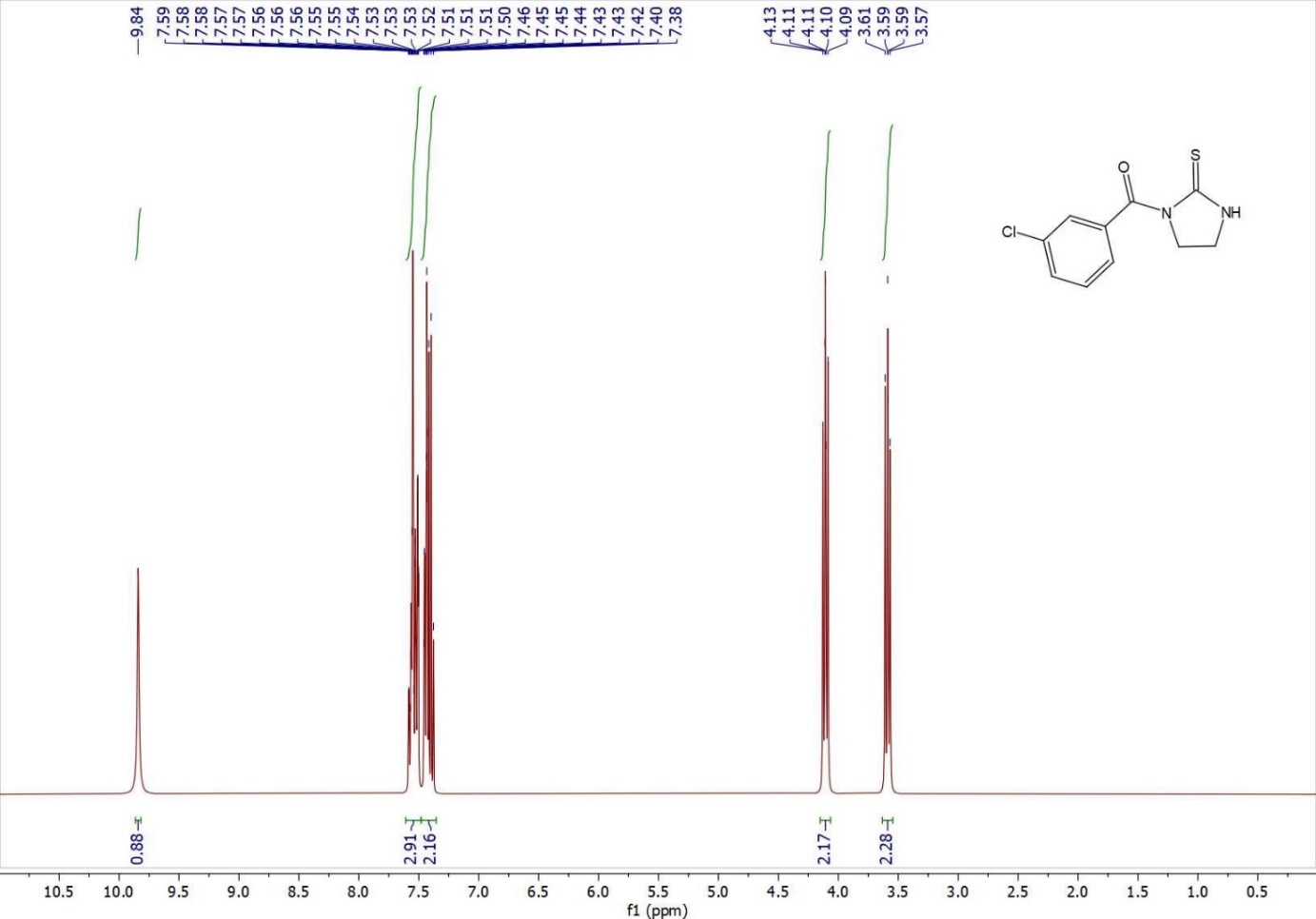
**

**Fig. S9** ^1^H NMR (400 MHz, d_6_-DMSO) of compound **2e**

**
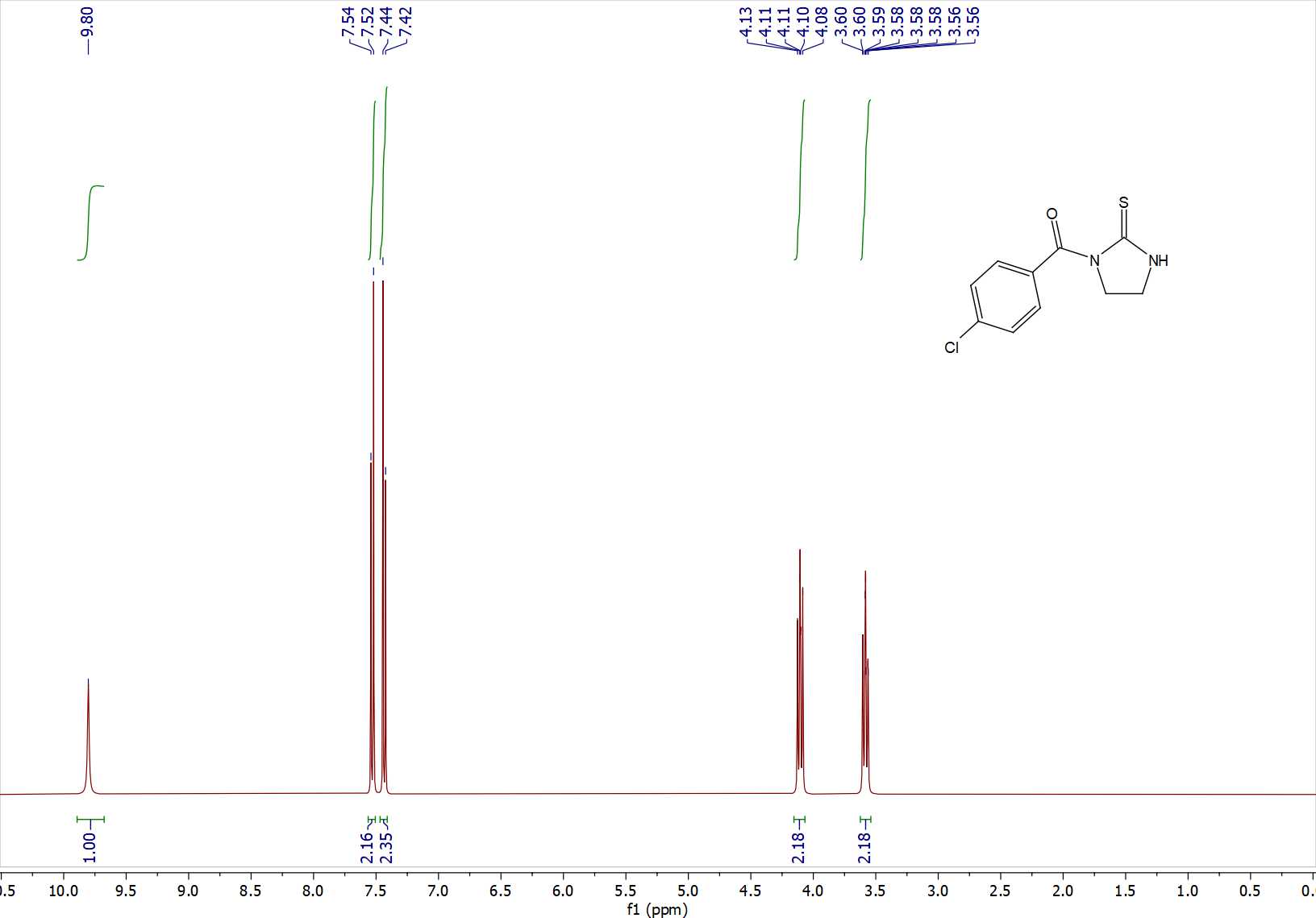
**

**Fig. S10** ^1^H NMR (400 MHz, d_6_-DMSO) of compound **2f**

**
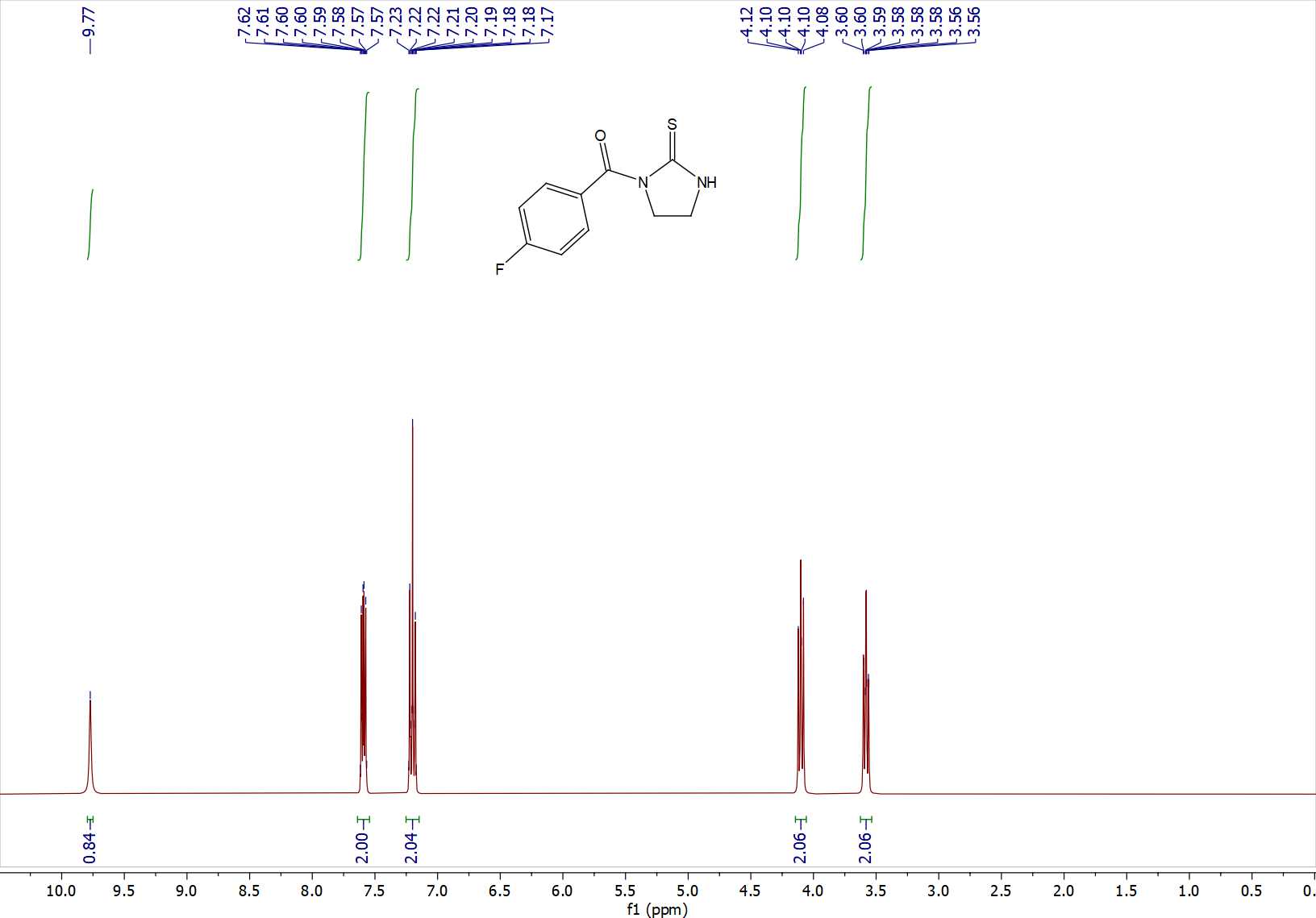
**

**Fig. S11** ^1^H NMR (400 MHz, d_6_-DMSO) of compound **2g**

**
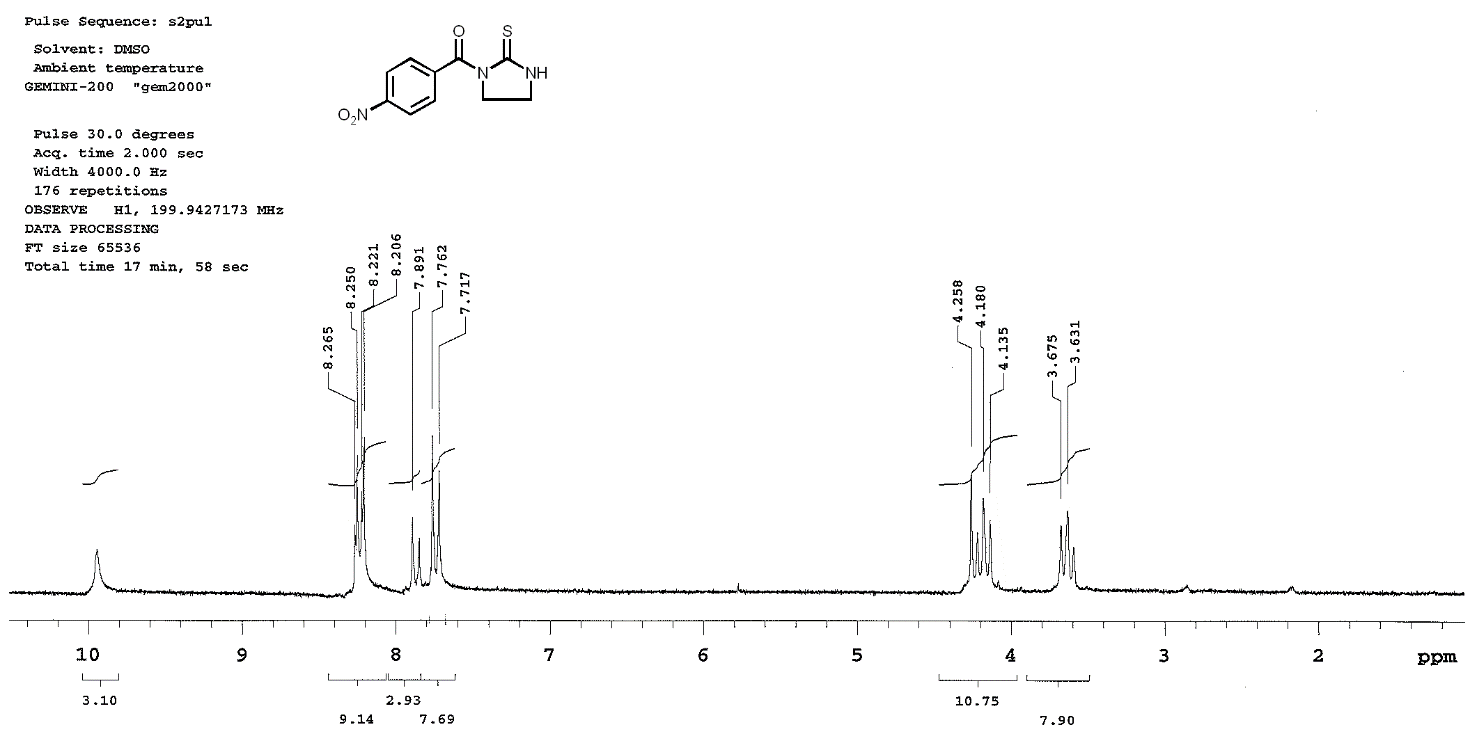
**

**Fig. S12** ^1^H NMR (200 MHz, d_6_-DMSO) of compound **2h**

**Fig. S13** ^1^H NMR (400 MHz, d_6_-DMSO) of compound **2j**

**Fig. S14** ^13^C NMR (101 MHz, DMSO-D_6_) of compound **2j**

**
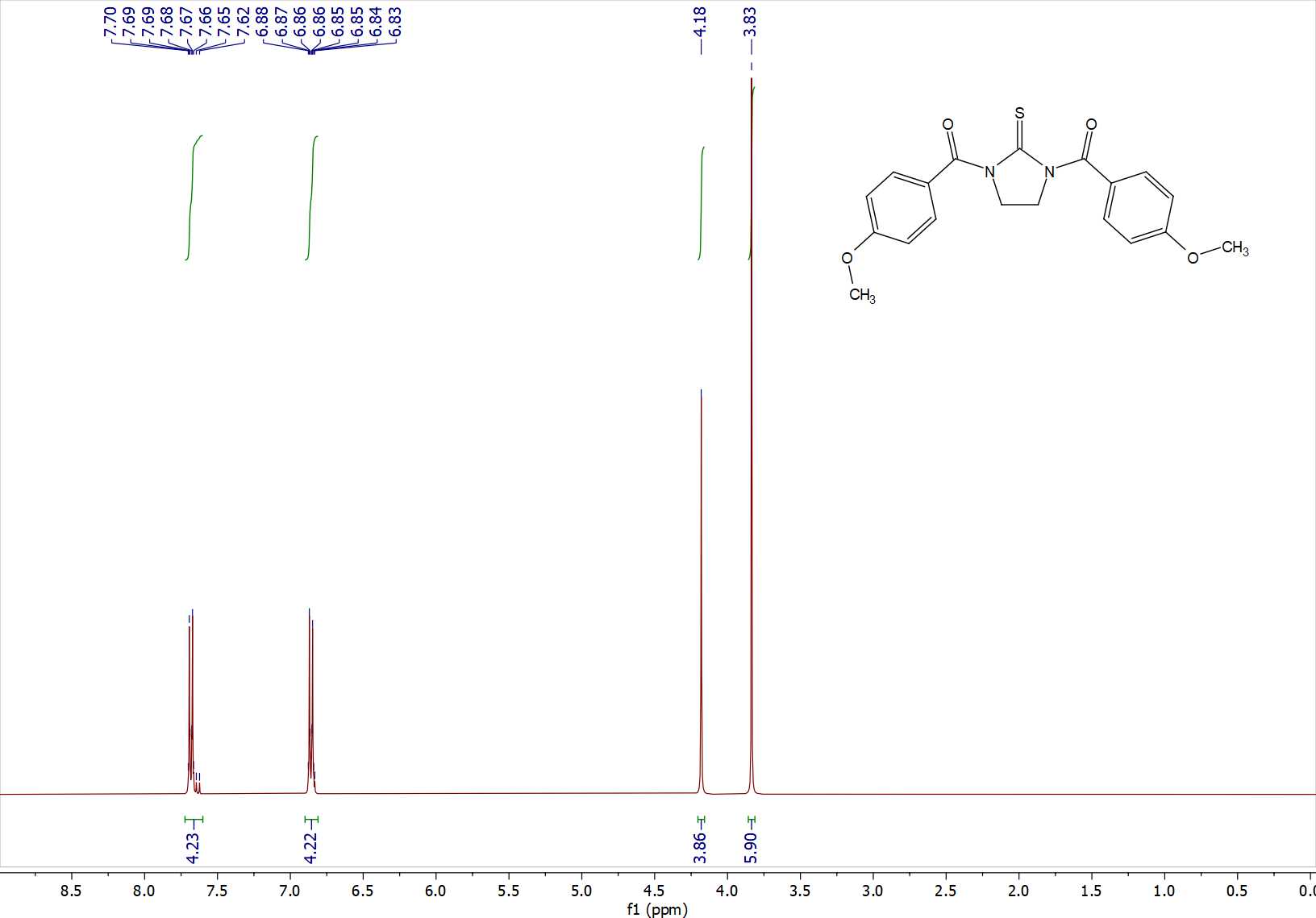
**

**Fig. S15** ^1^H NMR (400 MHz, CDCl_3_) of compound **3i**

**
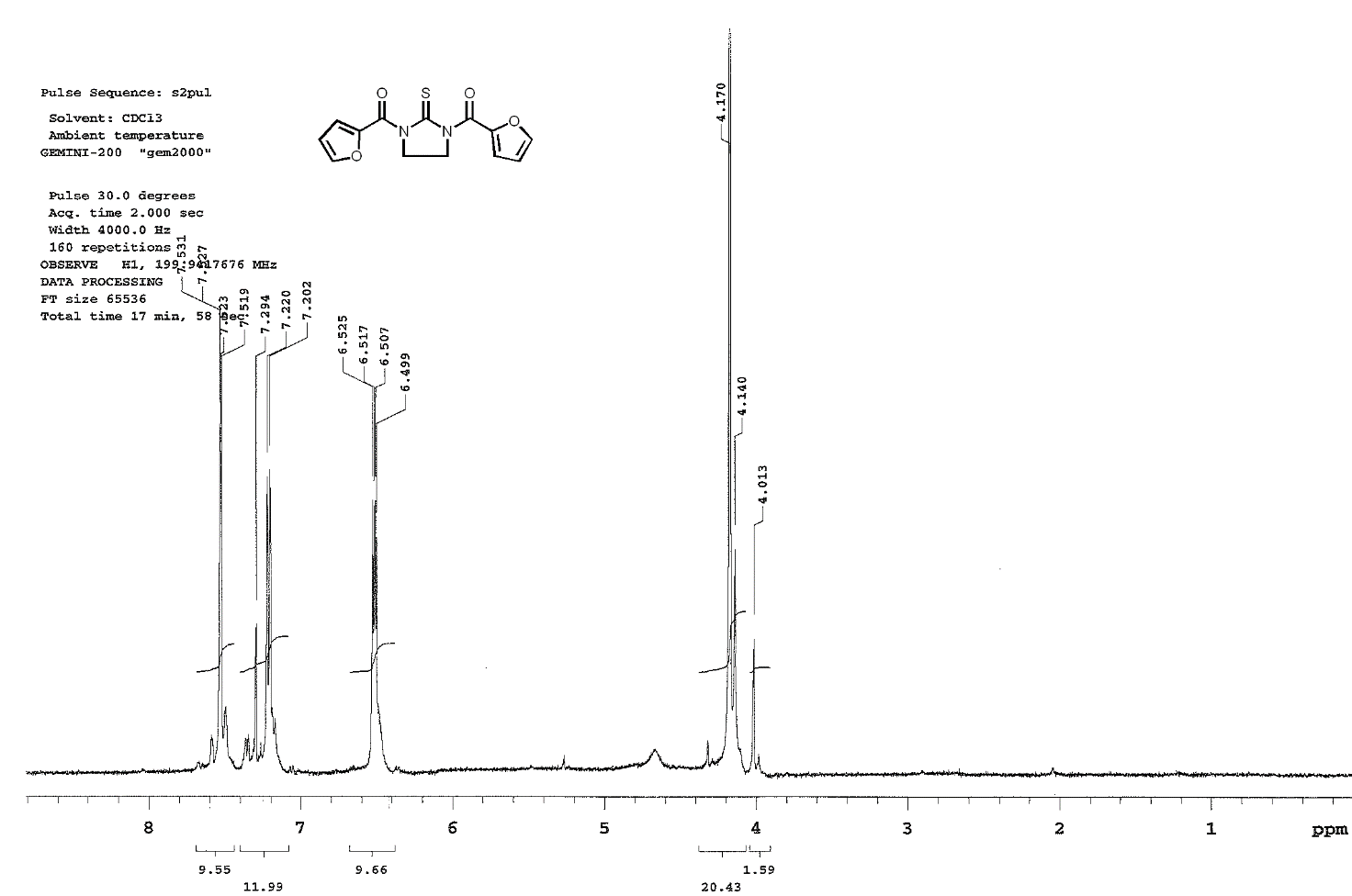
**

**Fig. S16** ^1^H NMR (200 MHz, CDCl_3_) of compound **3k**


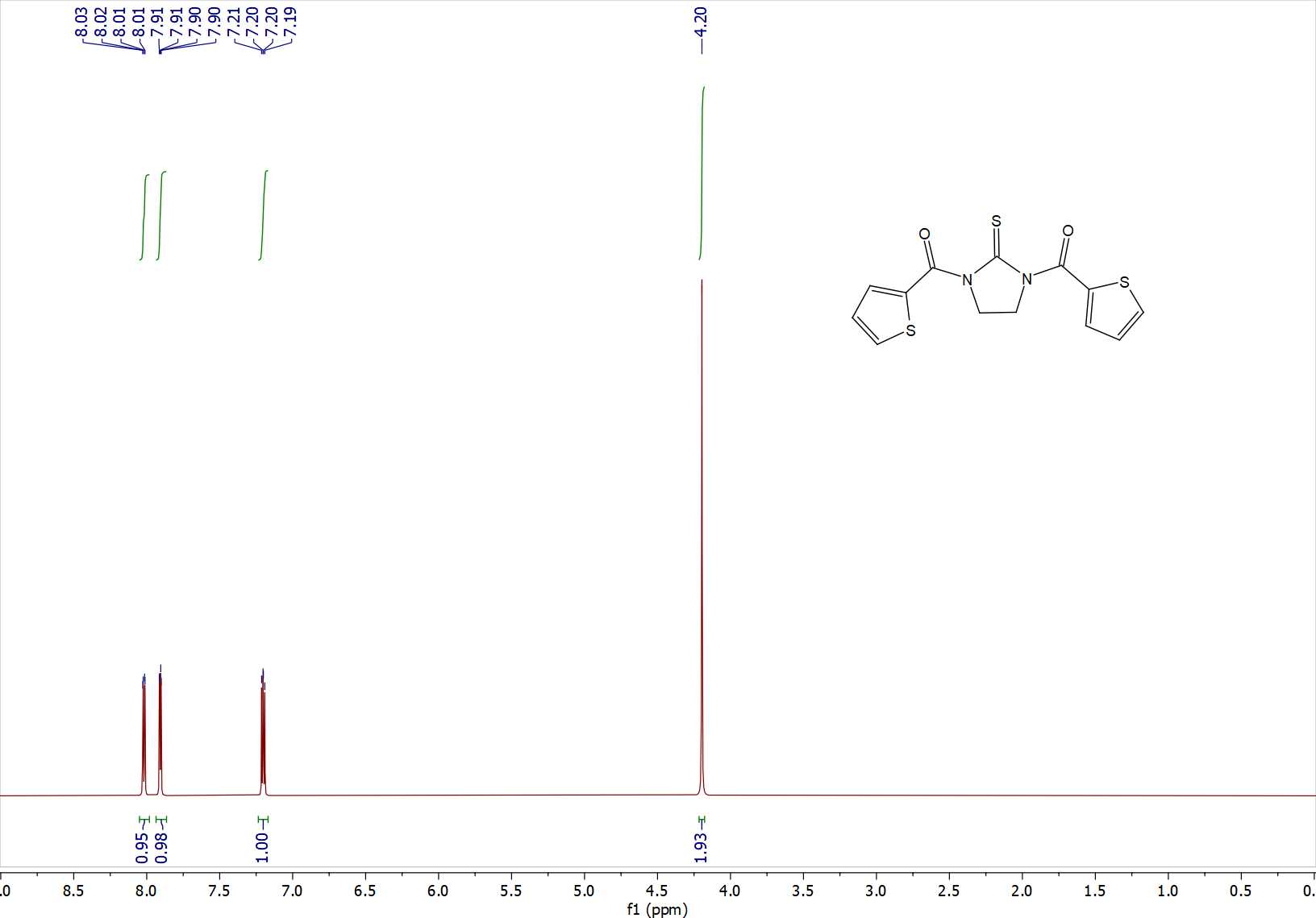


**Fig. S17** ^1^H NMR (400 MHz, DMSO-D6) of compound **3l**

**
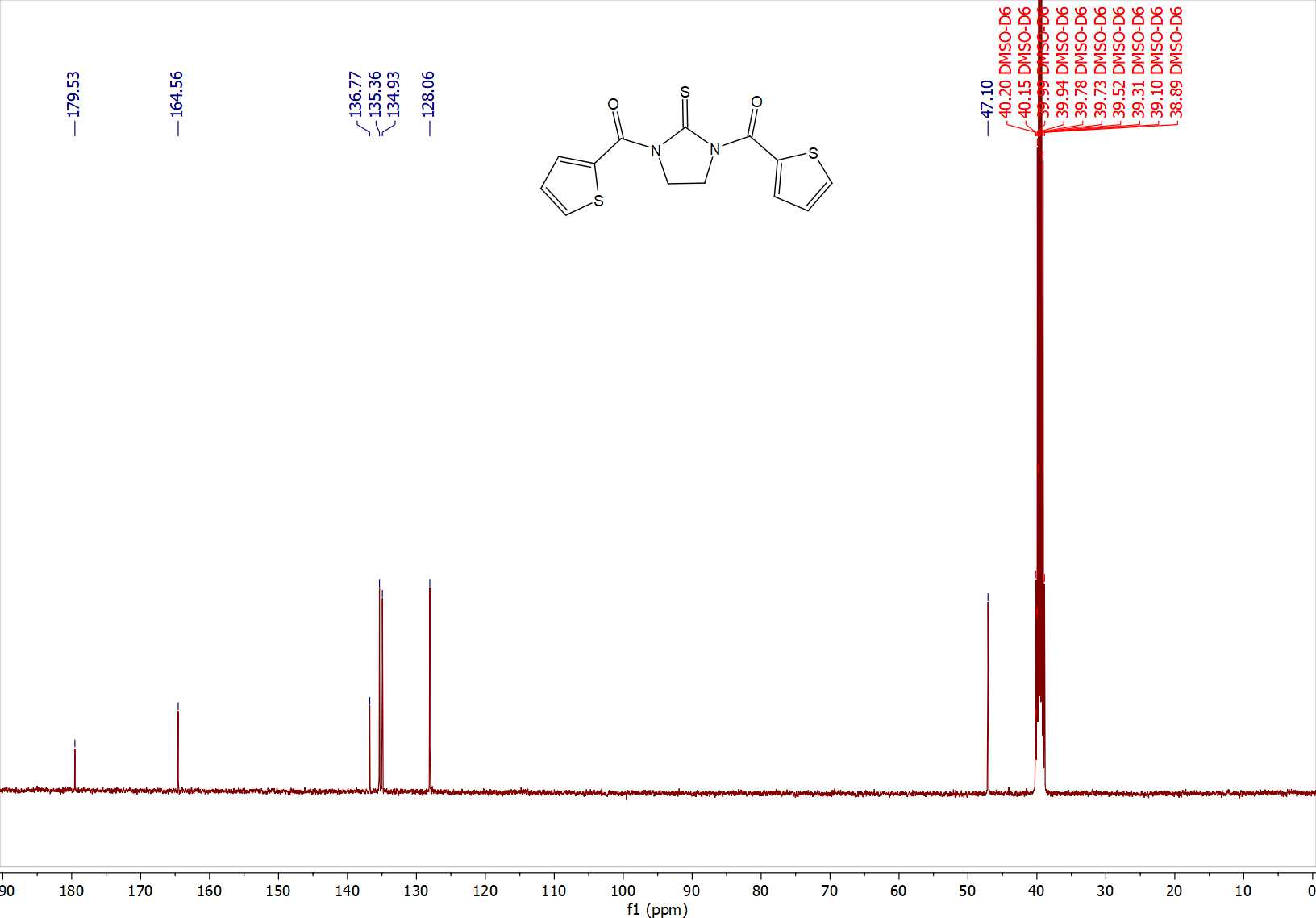
**

**Fig. S18** ^13^C NMR (101 MHz, DMSO-D6) of compound **3l**

**Fig. S19** ^1^H NMR (400 MHz, CDCl_3_) of compound **4**

**Fig. S20** ^13^C NMR (101 MHz, CDCl_3_) of compound **4**


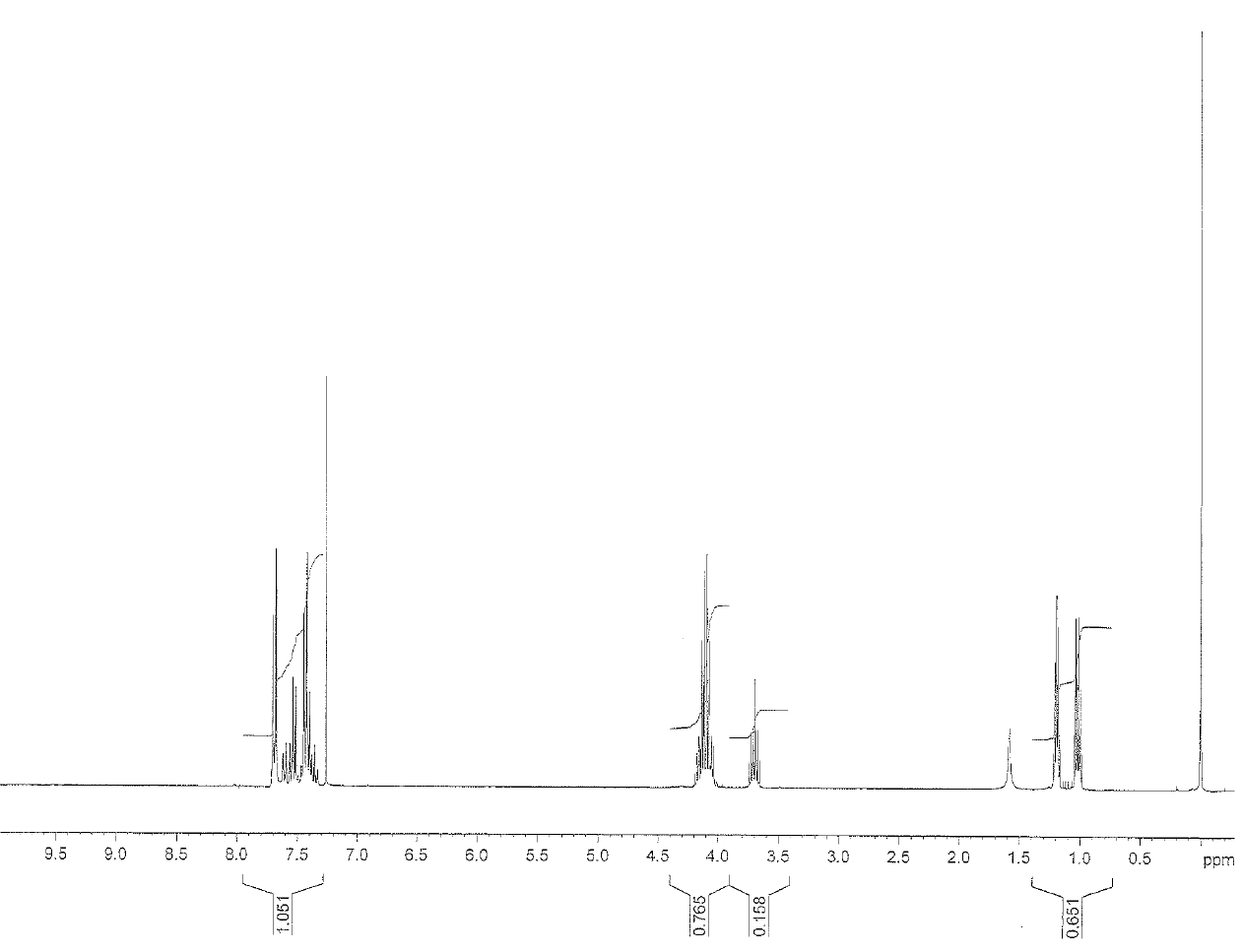


**Fig. S21** ^1^H NMR (300 MHz, CDCl_3_) of compound **5**


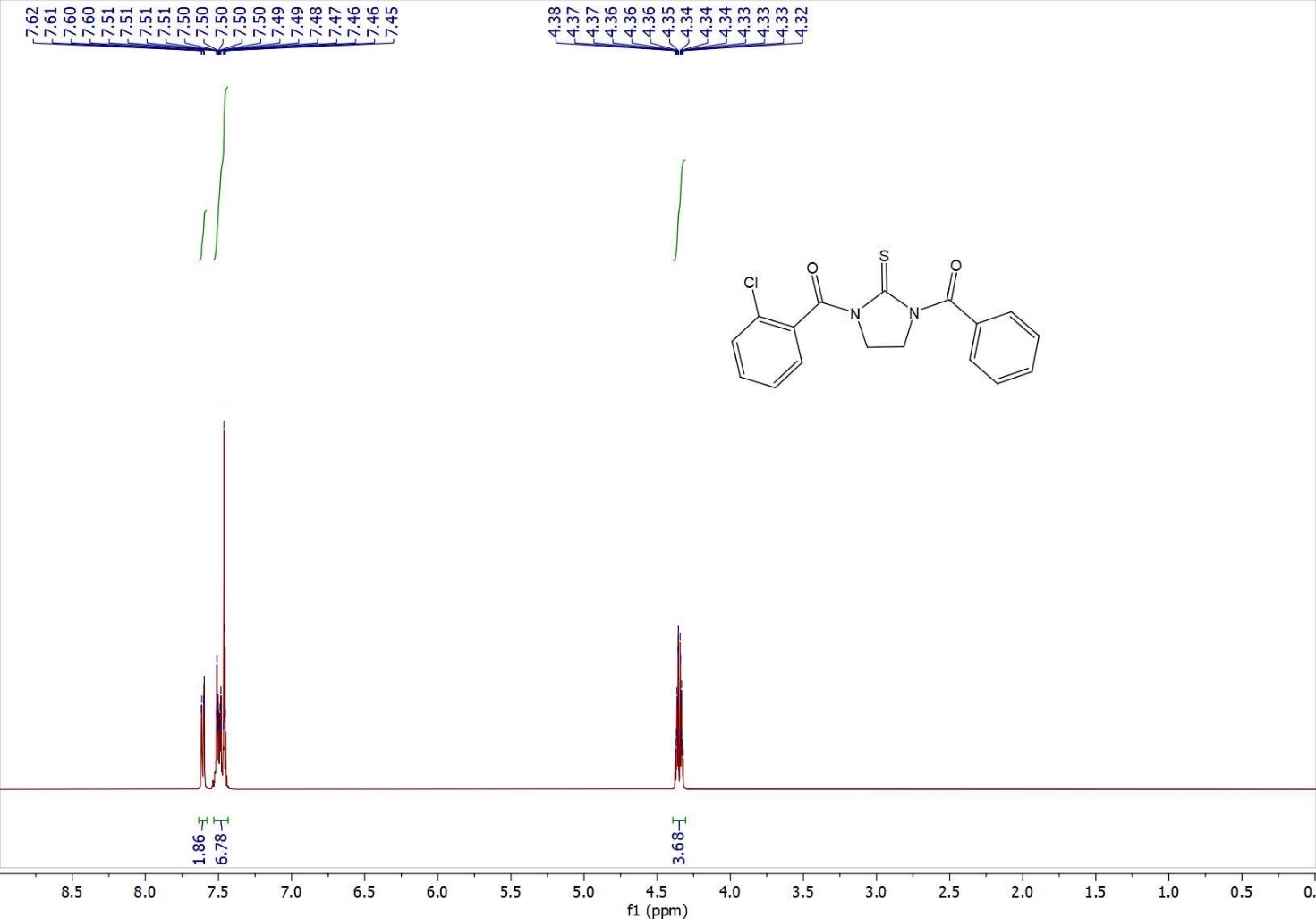


**Fig. S22** ^1^H NMR (400 MHz, DMSO-D6) of compound **6**


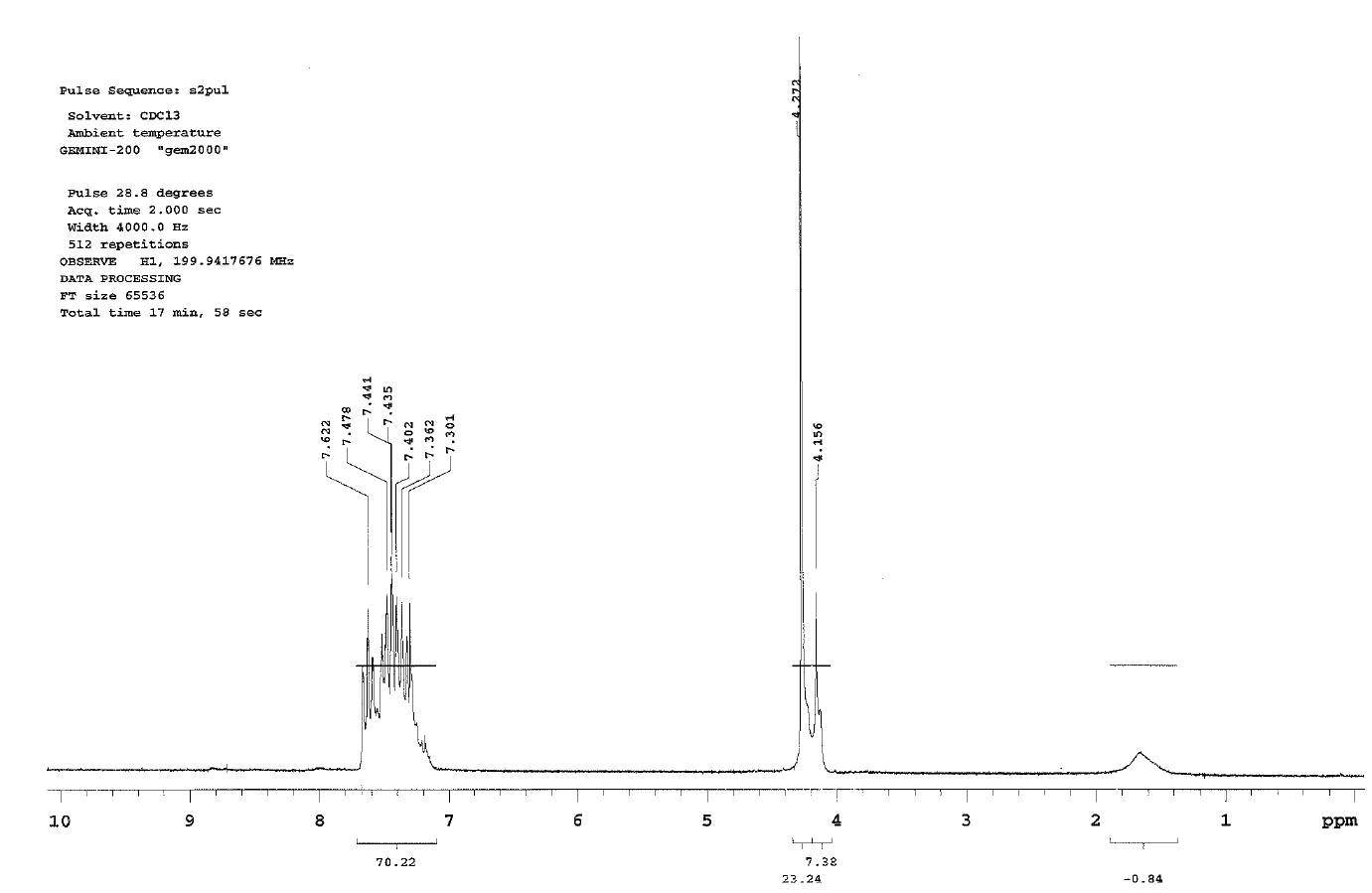


**Fig. S23** ^1^H NMR (200 MHz, CDCl_3_) of compound **7**

**Fig. S24** ^1^H NMR (400 MHz, CDCl_3_) of compound **8**

 **Fig. S25** ^13^C NMR (101 MHz, CDCl_3_) of compound **8**

**Fig. S26** ^1^H NMR (400 MHz, CDCl_3_) of compound **9**

**Fig. S27** ^13^C NMR (101 MHz, CDCl_3_) of compound **9**

**Fig. S28** ^1^H NMR (400 MHz, CDCl_3_) of compound **10**

**Fig. S29** ^13^C NMR (101 MHz, CDCl_3_) of compound **10**


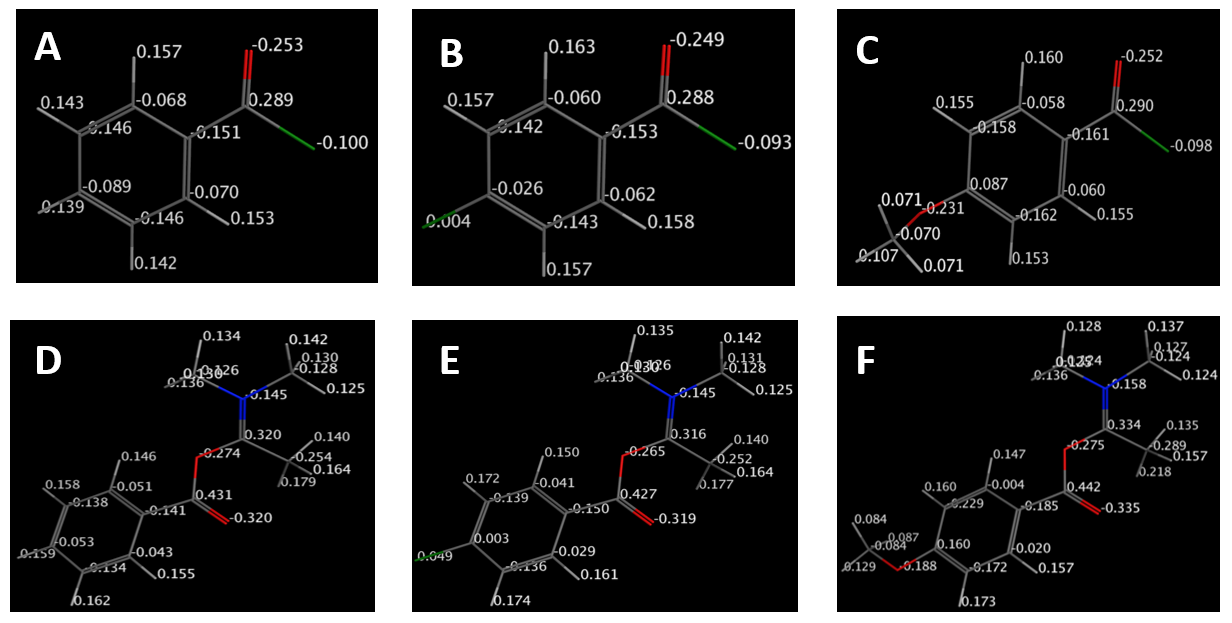


**Fig. S30** Partial charge distribution of benzoyl chloride (A), 4-chlorobenzoyl chloride (B), 4-anisoyl chloride (C) and their corresponding intermediates II (D-F).
